# Superset Versus Traditional Resistance Training Prescriptions: A Systematic Review and Meta-analysis Exploring Acute and Chronic Effects on Mechanical, Metabolic, and Perceptual Variables

**DOI:** 10.1007/s40279-025-02176-8

**Published:** 2025-02-04

**Authors:** Xing Zhang, Jonathon Weakley, Hansen Li, Zhaoqian Li, Amador García-Ramos

**Affiliations:** 1 Department of Physical Education and Sport, Faculty of Sport Sciences, University of Granada, Granada, Spain; 2 School of Behavioural and Health Sciences, Australian Catholic University, Brisbane, QLD Australia; 3 Sports Performance, Recovery, Injury and New Technologies (SPRINT) Research Centre, Australian Catholic University, Brisbane, QLD Australia; 4 Carnegie Applied Rugby Research (CARR) Centre, Carnegie School of Sport, Leeds Beckett University, Leeds, UK; 5 School of Physical Education, Sichuan Agricultural University, Ya’an, China; 6 Department of Sports Sciences and Physical Conditioning, Faculty of Education, Universidad Católica de La Santísima Concepción, Concepción, Chile

## Abstract

**Background:**

Supersets are a time-efficient resistance training (RT) method that involve the sequencing of two exercises with little or no rest between them. However, despite their common implementation during RT, a comprehensive and quantitative review is still lacking.

**Objectives:**

The primary aim of this systematic review and meta-analysis was to compare the acute and chronic effects of superset and traditional set prescriptions on mechanical, metabolic, and perceptual variables. We also aimed to conduct subgroup analyses to determine the effect of different types of supersets (agonist–antagonist, similar biomechanical, and alternate peripheral supersets).

**Methods:**

A systematic literature search was conducted in PubMed, Web of Science, Embase, and EBSCO databases from inception to 10 February 2024. Studies written in English and meeting our inclusion criteria were included. Pooled meta-analysis and subgroup meta-analysis were performed using a random-effects model.

**Results:**

Nineteen studies involving 313 participants were included. Although there was considerable variance in certain outcomes, our estimated effects suggested that, compared with traditional set prescription, supersets allow for (1) a similar total number of repetitions [standardized mean differences (SMD) =  − 0.03; *p* = 0.92] and volume load (SMD = 0.05; *p* = 0.86) with a shorter session duration and increased training efficiency (SMD = 1.74; *p* = 0.01); (2) higher blood lactate concentration during (SMD = 0.94; *p* = 0.03) and after (SMD = 1.13; *p* < 0.01) RT; (3) higher energy cost during RT (SMD = 1.93; *p* = 0.04); (4) similar creatine kinase concentration after RT (SMD = 0.22; *p* = 0.36), surface electromyography (SMD = 0.01; *p* = 0.98), acute muscle swelling (SMD = − 0.28; *p* = 0.36) and blood pressure (systolic blood pressure [SMD = 0.08; *p* = 0.71], diastolic blood pressure [SMD = − 0.05; *p* = 0.85], and mean arterial pressure [SMD = − 0.03; *p* = 0.88]); (5) higher rating of perceived exertion (SMD = 0.77; *p* = 0.02) and similar perceived recovery (SMD = 0.32; *p* = 0.33); and (6) similar chronic adaptations in maximal strength (SMD = 0.10; *p* = 0.36), strength endurance (SMD = 0.07; *p* = 0.81), and muscle hypertrophy (SMD =  − 0.05; *p* = 0.87). The subgroup analysis revealed that utilizing agonist–antagonist supersets leads to a significant increase in the number of repetitions that are able to be completed compared with traditional sets (SMD = 0.68; *p* = 0.01). Similar biomechanical supersets led to less volume load (SMD =  − 1.08; *p* < 0.01) compared with traditional sets.

**Conclusions:**

Supersets provide a time-efficient alternative to traditional RT, reducing session duration without compromising training volume, muscle activation, perceived recovery, or chronic adaptations in maximal strength, strength endurance, and muscle hypertrophy. Thus, supersets can be effectively implemented by athletes with busy schedules and RT enthusiasts whose main barrier to exercise is time. However, it should be noted that supersets generally induce higher internal loads, more severe muscle damage, and increased perceived exertion, potentially necessitating extended recovery times between sessions. Additionally, superset RT may have a similar potential to traditional RT in eliciting post-exercise hypotension. Regarding different types of supersets, agonist–antagonist supersets are more suitable for maintaining training volume, while similar biomechanical supersets concentrate stimulation on the same muscle group, compromising volume load.

**Protocol Registration**: The original protocol for this review was prospectively registered with the International Prospective Register of Systematic Reviews (PROSPERO) in December 2023 (CRD42023491533).

## Key Points

**Table:** 

Supersets can enhance training efficiency by reducing training duration without compromising training volume, muscle activation, or perceived recovery.
Compared with traditional sets, supersets induce higher internal loads, more severe muscle damage, and increased perceived exertion.
Supersets can achieve comparable chronic adaptations to traditional set prescriptions in maximal strength, strength endurance, and muscle hypertrophy.

## Introduction

Resistance training (RT) is widely used as a method for enhancing maximal strength [1, 2], jumping [3, 4], sprinting [5], and change of direction [6]. Moreover, RT has been proven to be effective in preventing and rehabilitating sports injuries by contributing to the strengthening of ligaments, tendons, and connective tissue within the muscle [7]. RT also plays a pivotal role in improving physical and mental health while enhancing overall quality of life [8–12]. However, to maximize the benefits of RT, it is essential to carefully consider the prescription of various training variables, such as training intensity, lifting velocity, velocity loss, training frequency, interset rest, training volume, and set structures [13, 14]. Among these factors, set structure is an important variable in modulating the effects of RT, and numerous studies have explored how different RT set structures affect immediate responses and long-term adaptations [15, 16].

Resistance training often involves performing multiple sets of an exercise with interset rest periods. Once all sets of one exercise are completed, the next exercise is performed. This “traditional” set structure has been widely adopted in training programs [17–20]. However, this approach requires substantial time to be spent resting between sets and exercises [21–23]. With lack of time commonly reported as one of the biggest barriers to exercise [24–26], training efficiency is a crucial consideration for the general population and athletes often must balance competing demands within tight schedules [27]. Therefore, more time-efficient RT methods could boost exercise participation among the general public and support the physical and social demands of athletes.

To develop more time-efficient RT methods, strength and conditioning experts have explored various approaches [15, 16, 28]. Specifically, superset RT, which involves the consecutive performance of two exercises with little or no rest between them, is considered a potential alternative. After completing all exercises in a superset, a rest period is taken before starting the next superset. This approach significantly reduces total rest time, thus increasing the overall efficiency of RT sessions. Previous studies have indicated that supersets can shorten training duration by approximately 50% compared with traditional set prescriptions [23, 28–30]. However, the shorter recovery periods in supersets may result in increased metabolic disturbance and fatigue, potentially leading to different training adaptations [29, 31, 32]. Furthermore, it is important to note that superset sequencing encompasses various forms, such as agonist–antagonist, similar biomechanical, and alternate peripheral supersets [31]. Although these superset structures are similar, they differ in the exercises used. For example, similar biomechanical supersets require exercises targeting the same muscle group (e.g., barbell bench press and dumbbell press) [31], whereas alternate peripheral supersets focus on pairing upper- and lower-body exercises (e.g., bench press and back squat) [31]. These differences can lead to varying outcomes.

To date, numerous experimental studies have been conducted to explore the distinctions between superset and traditional set prescriptions [28, 29, 31–33]. However, they have not yielded a unified conclusion and, at times, have even presented contradictory results. Therefore, a comprehensive systematic review and meta-analysis is still warranted to elucidate the differences between superset and traditional set prescriptions. Consequently, the primary aim of the current systematic review and meta-analysis was to establish and compare the acute and chronic effects of superset and traditional set prescriptions on mechanical, metabolic, and perceptual variables. Additionally, we examine how different superset structures vary in their acute and chronic effects.

## Methods

### Registration of Systematic Review and Meta-analysis Protocol

The current systematic review and meta-analysis adhered to the guidelines outlined in the Cochrane Handbook for Systematic Reviews of Interventions, version 5.1.0, and followed the Preferred Reporting Items for Systematic Reviews and Meta-Analysis (PRISMA) 2020 checklist [34]. The original protocol for this review was prospectively registered with the International Prospective Register of Systematic Reviews (PROSPERO) in December 2023 (CRD42023491533).

### Information Sources and Search Strategy

A systematic literature search was performed using the following English electronic databases: PubMed, Web of Science, Embase, and EBSCO. The search period extended from the inception of each database to 10 February 2024. The following syntax was adapted for each database and applied to the title, abstract, and keyword search fields: (“resistance training” OR “weight training” OR “strength training”) AND (“superset” OR “paired set” OR “compound set” OR “agonist antagonist” OR “complex set” OR “alternate”). In the subsequent phase of the search, the reference lists of the review studies identified in the initial search were screened. Additionally, studies that met the inclusion criteria were used to further identify potential literature through the “Related articles” feature in Google Scholar. Free-text terms were applied based on pilot searches to strike an appropriate balance between search sensitivity and precision, without the use of controlled vocabulary (e.g., medical subject headings). The strategy exclusively encompassed terms related to the intervention and did not include population/subject information. Abstracts, letters to the editor, commentaries, proceedings, and theses were excluded.

### Eligibility Criteria

A PICO principle was applied to build eligibility criteria for including/excluding studies as follows:

- P (Population): Healthy individuals without known medical conditions or injuries;

- I (Intervention): RT with superset structures were considered. Excluded were combinations of plyometric training and RT, as this combination is commonly referred to as complex training, which falls into a different domain;

- C (Comparison): Comparisons between superset and traditional set prescriptions during and/or after intervention;

- O (Outcomes): Acute indicators including mechanical, metabolic, and perceptual responses, as well as long-term adaptations including muscle hypertrophy, maximal strength, and athletic performance indicators.

Only studies that underwent peer review and were published in English were considered [35, 36]. Finally, traditional set prescription was defined as the execution of multiple sets of one RT exercise with consistent interset rest periods. Additionally, after completing all sets of one RT exercise, the next RT exercise is performed following a predefined rest period. Superset prescription was defined as the consecutive performance of two RT exercises, with either no rest or little rest (e.g., traveling between exercises) between them. After completing the two RT exercises included in one superset, a rest period is taken before commencing the next superset. As such, superset prescription should include at least two sets of each exercise. For subgroup analyses, supersets were divided according to the groups defined by Weakley et al. [31]: (1) agonist–antagonist supersets, which involved one RT exercise targeting a specific body part and another targeting its antagonist part (e.g., bench press and bench row); (2) similar biomechanical supersets, which involved two RT exercises targeting the same muscle group (e.g., bench press and dumbbell bench press); and (3) alternate peripheral supersets, which involved one RT exercise targeting lower-body muscles and another targeting upper-body muscles (e.g., squat and bench press).

### Study Selection

The assessment of study eligibility was independently conducted by two reviewers (XZ and HSL). All records were downloaded into Endnote X8 (Clarivate Analytics, Philadelphia, PA, USA), and duplicates were removed based on author, title, and publication year before the screening process. Subsequently, the titles and abstracts were screened to determine initial eligibility. Following this, full texts of the remaining records were retrieved and assessed for eligibility. Any discrepancies throughout the study selection process were resolved by discussion between two reviewers or, if necessary, by the judgment of a third reviewer (AGR).

### Data Items and Data Collection Process

From the studies that met the inclusion criteria, the following data were extracted into an Excel spreadsheet: (1) study identification information; (2) study design; (3) sample size; (4) participants’ age, strength level, sex, and training experience; (5) RT prescription details for superset and traditional set structures; and (6) means and standard deviation for relevant outcome measures. For studies in which the authors reported the data exclusively in figures, the GetData Graph Digitizer 2.26 software (GetData Software Pty Ltd, Kogarah, NSW, Australia) was employed to extract data from the figure. If insufficient data were provided in the original studies, the authors of those studies were contacted via email. The data extraction process was independently conducted by two authors. Any discrepancies throughout the data collection process were resolved by discussion between two authors (XZ and HSL) or, if necessary, by the judgment of a third author (AGR).

### Assessment of Bias and Evidence Quality

A risk of bias assessment was conducted using a modified version of the Cochrane Collaboration's tool for evaluating the risk of bias in eligible studies [37]. The modifications involved the removal of the performance bias item and the addition of outcome assessment bias, effort bias, and familiarization bias items. The performance bias item was removed because it was considered impossible to successfully blind participants and personnel in supervised exercise intervention studies [38–40]. Outcome assessment bias held particular significance in this review, focusing on the reliability and appropriateness of the equipment or instruments used to evaluate the outcomes of interest in each study [16]. An included study was considered to be at low risk of outcome assessment bias if prior research had demonstrated the reliability of the equipment or instruments employed to record mechanical, metabolic, and perceptual variables. Effort bias was also a critical consideration in this review, as varying effort levels could lead to differences in mechanical, metabolic, or perceptual measures [16]. An included study was considered as a low risk of effort bias if the authors clearly stated that all participants performed the RT exercises with maximal effort. Familiarization bias emerged as another significant factor affecting the implementation of exercise interventions, as participants’ familiarity with study protocols could influence exercise performance [16]. An included study was considered as a low risk of familiarization bias if the authors clearly indicated that a familiarization session had occurred or if the participants regularly engaged with the RT exercises used in the study. The risk of bias assessment was based on the information reported in the published papers, rather than information provided by the authors. The risk of bias assessment was independently conducted by two authors (XZ and HSL). Any discrepancies throughout the assessment of the bias process were resolved by discussion between two authors or, if necessary, by the judgment of a third author (AGR).

The evidence quality of assessment was performed using the Grading of Recommendations Assessment, Development, and Evaluation (GRADE) system. The overall quality of the evidence synthesis was rated as high and then downgraded by one level to moderate, low, or very low based on the following limitations: (1) risk of bias: if more than 50% of the studies within a specific meta-analysis had more than one risk of bias item assessed as high risk of bias; (2) imprecision: if the total sample size was less than 100 participants; (3) inconsistency: in the presence of high statistical heterogeneity; (4) indirectness: when the interventions under investigation were indirectly compared in a population not directly relevant to the study.

### Statistical Analysis

When two or more studies reported on the same outcome, a random-effects meta-analysis for each review outcome was conducted using the Stata 18 software (StataCorp LLC, Texas, USA). Model parameters were calculated using the maximum likelihood method, and the observations were weighted by the inverse of the sampling variance. The pooled synthesis between the superset and traditional set prescriptions was calculated using standardized mean differences (SMD) with a 95% confidence interval (CI). Given the small sample sizes in most outcomes, Hedges’ *g* correction was applied [41]. Additionally, 95% prediction intervals (PIs) were computed to estimate the potential range of effect sizes in future studies, reflecting not only the uncertainty of effect sizes within the current studies, but also accounting for the impact of heterogeneity across studies. For acute indicators, the SMD, 95% CI, and 95%PI were calculated based on the mean and standard deviation. For chronic indicators, the SMD, 95%CI, and 95% PI were calculated based on the change in mean and standard deviation from pre- (baseline) to post-intervention. The change in standard deviations was calculated following the guidelines provided in the Cochrane Handbook for Systematic Reviews of Interventions [42]. Since no studies reported any correlations between pre- and post-intervention, a conservative correlation coefficient of 0.5 was used to ensure the inclusion of the maximum number of studies in the meta-analysis [43]. The magnitude of the SMD was interpreted as follows: small (0.20–0.49), moderate (0.50–0.79), and large (> 0.80) [38]. Subgroup analyses were performed if two or more studies reported the same outcome with the same type of superset. The heterogeneity of the studies was assessed using the *I*^2^ statistic. The *I*^2^ statistic represents the percentage of total variation in estimated effects across studies due to heterogeneity rather than chance, and *I*^2^ ≥ 75% was regarded as high heterogeneity [44, 45]. Sensitivity analysis and publication bias were not assessed because there were only a small number of studies included in most meta-analyses and all studies were of a similar size [42]. In all analyses, *p* values < 0.05 were considered statistically significant.

To reduce bias induced by duplicate inclusions, only the maximum average between superset and traditional set structures was utilized when multiple outcomes were reported in a study. For instance, in the study by Weakley et al. [28], blood lactate concentrations during the intervention were reported after 6 and 12 sets. Only the measurement after 12 sets was used because it provided the maximum blood lactate concentration during the intervention. In addition, the study by Wallace et al. [33] implemented the same intervention in two different sessions. The outcomes from these separate interventions were independently incorporated into our meta-analysis. Finally, to accurately capture the variations between superset and traditional set structures, studies with a fixed number of repetitions were excluded from the analysis of training volume and efficiency, as the predetermined and controlled total volume limits meaningful comparisons between training protocols.

## Results

### Study Selection

**Fig. 1:**
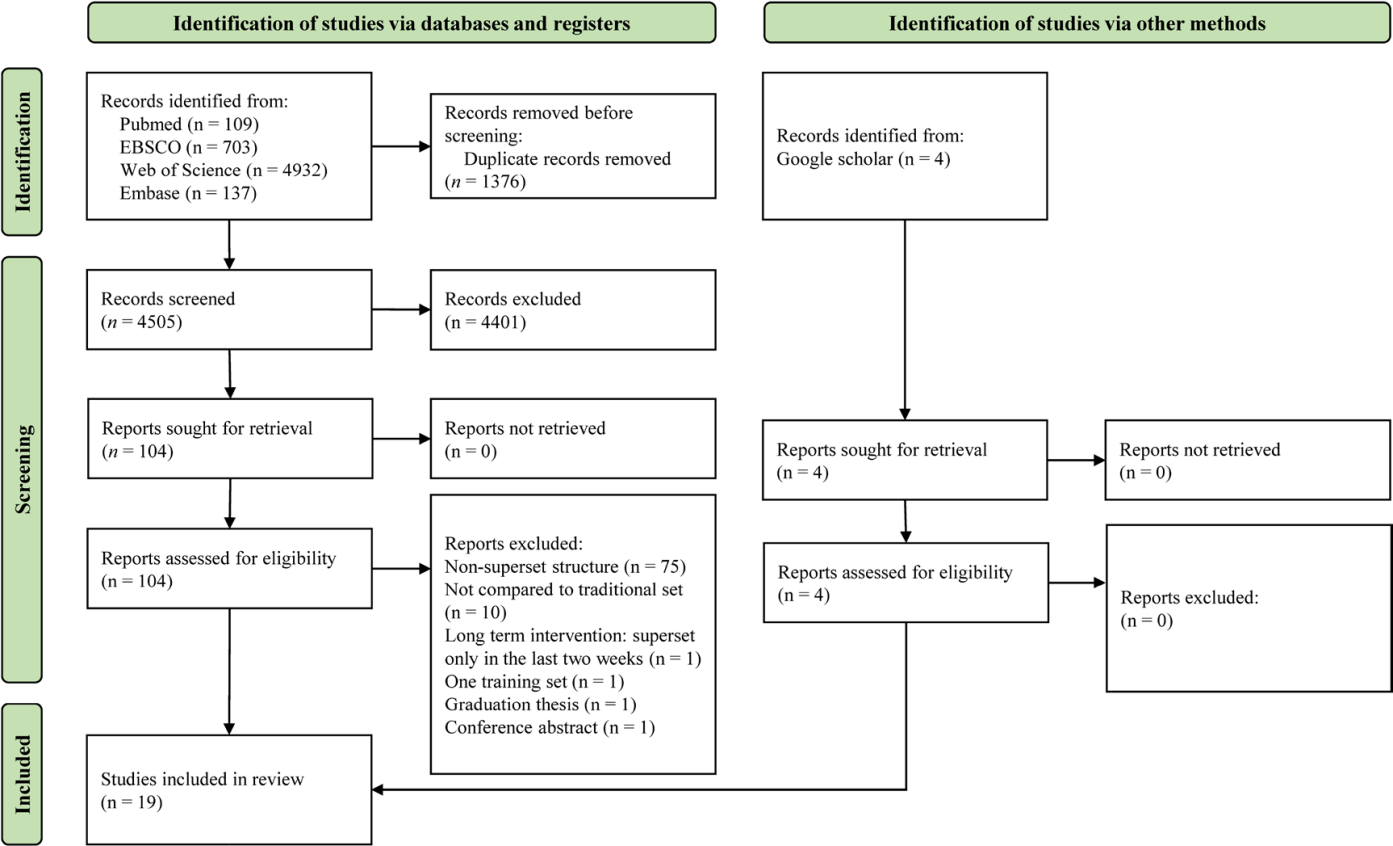
The study selection flow diagram of included and excluded research

The flow diagram of the study selection process is depicted in Fig. 1. The initial database search yielded a total of 5881 studies. After that, 1376 studies were excluded due to duplication and 4401 studies were excluded based on title and abstract screening. In the subsequent phase of the selection, four studies were identified. Consequently, 108 studies were assessed for eligibility. Based on the assessment results, 89 studies were excluded for reasons including non-superset structure, lack of comparison to traditional set, inappropriate training design, being a graduation thesis, or being a conference abstract. Eventually, 19 studies were included in this review.

### Study Characteristics

A total of 19 studies, involving 313 participants, were included in this systematic review and meta-analysis. Most studies (*n* = 16) exclusively compared the acute effects of superset and traditional set structures: only three studies compared their chronic effects [32, 46, 47]. In terms of participants, most participants were male (*n* = 232), with only 66 females included, while one study did not report the participants’ sex (*n* = 15) [48]. In addition, 15 studies reported that their participants had RT experience of at least 0.5 years. Two studies included participants who took part in soccer [49] or Brazilian jiu-jitsu [30], while the remaining two studies used physically active participants not affiliated with any specific sport [47, 50]. Regarding training design, 11 studies indicated that all sets were performed to failure. Seven studies used a submaximal number of repetitions [28, 29, 31, 32, 47, 48, 50], and one study mentioned that all sets were performed to failure, but if repetitions exceeded ten repetitions in a set, the set would be terminated [33]. The main outcomes included in this review were total number of repetitions, volume load [total number of repetitions × load (kg)], session duration, training efficiency [representing the amount of work completed within a given unit of time, calculated as volume load/session duration (kg min^−1^)], blood lactate (blood lactate concentration during and after intervention), creatine kinase (creatine kinase concentration after intervention), energy cost (energy consumption per minute in kJ or kcal during intervention), surface electromyography (percentage of maximum voluntary contraction), acute muscle swelling (acute changes in muscle size after intervention), blood pressure (systolic blood pressure, diastolic blood pressure, and mean arterial pressure after intervention), rating of perceived exertion, perceived recovery, one repetition maximum (1 RM), maximal number of repetitions (MNR), and muscle cross-sectional area (muscle CSA; chronic intervention induced changes in physiological cross-sectional area). It is important to note that one study did not report any outcomes the same as those mentioned above [48], and thus did not meet our meta-analysis criteria (two or more studies reporting on the same outcome). A more detailed description of the included studies can be found in Table 1.

**Table 1:** Characteristics of the studies included in the review

Study	Participants’ information		Intervention design			Outcome
	Sample size (sex); Age	Training experience Strength levels	Sets × repetitions Rest time	Load	Exercise	
Garcia-Orea et al. [29]	19 (M) 24.0 ± 5.0 years	RT: 0.5–3 years SQ: 93.6 ± 19.1 kg (1 RM) BP: 72.4 ± 12.4 kg (1 RM)	AS: 3 sets × VL 15% and 20% Intraset rest: 45 s Interset rest: 2 min TS: 3 sets × VL 15% and 20% Interset rest: 3 min	55–70% 1 RM	Smith SQ Smith BP	Total number of repetitions Session duration
Andersen et al. [23]	29 (15 F + 14 M) 27.2 ± 7.2 years	RT: At least 1 year BP: 70.7 ± 28.6 kg (1 RM) SQ: 91.5 ± 32.0 kg (1 RM) DL: 111.6 ± 39.1 kg (1 RM)	S: 3 sets × failure Intraset rest: Succession Interset rest: 2 min TS: 3 sets × failure Interset rest: 2 min	9 RM	DL BP SQ Seal row Flies Triceps extension Reverse flies Biceps curl	RPE Total number of repetitions Session duration
Antunes et al. [51]	12 (M) 24.0 ± 3.3 years	RT: At least 0.5 years Leg extension: 47 ± 14 kg (10 RM) Seated leg curl 90: 75 ± 12 kg (10 RM) Seated leg curl 60: 62 ± 10 kg (1 RM)	AAS: 3 sets × failure Intraset rest: < 30 s Interset rest: 1 min TS: 3 sets × failure Interset rest: 1 min Unbalance training volume	10 RM	Knee flexions Knee extensions	
Belo et al. [30]	12 (M) 24.5 ± 3.1 years	Brazilian jiu-jitsu: 3.5 ± 2.8 years BP: 53.0 ± 11.3 (10 RM) Romanian DL: 55.3 ± 11.4 kg (10 RM) Leg press: 217.1 ± 45.9 kg (10 RM) Lat pulldown: 64.0 ± 6.9 kg (10 RM)	AS: 3 sets × failure Intraset rest: Succession Interset rest: 2 min TS: 3 sets × failure Interset rest: 2 min	10 RM	Romanian DL BP Leg press Lat pulldown	Total number of repetitions Session duration Volume load Blood lactate Training efficiency RPE
Weakley et al. [31]	10 (M) 20.9 ± 0.6 years	RT: At least 2 years BP: 114.3 ± 10.3 kg (3 RM) SQ: 139.7 ± 27.9 kg (3 RM) Bent-over row: 101.1 ± 12.0 kg (3 RM) Dumbbell BP: 87.5 ± 11.6 kg (3 RM)	AAS/AS/SS: 3 sets × 10 Intraset rest: Succession Interset rest: 2 min TS: 3 sets × 10 Interset rest: 2 min Unbalance training volume	65% 3 RM	BP Dumbbell BP SQ Bent-over row	
Weakley et al. [28]	14 (M) 20.8 ± 1.2 years	RT: At least 2 years SQ: 141.1 ± 31.9 kg (3 RM) BP: 105.2 ± 15.2 kg (3 RM) Romanian DL: 143.2 ± 30.8 kg (3 RM) Dumbbell BP: 66.0 ± 8.6 kg (3 RM) Bent-over row: 95.0 ± 14.5 kg (3 RM) Upright row: 60.1 ± 6.9 kg (3 RM)	S: 3 sets × 10 Intraset rest: Succession Interset rest: 2 min TS: 3 sets × 10 Interset rest: 2 min	65% 3 RM	SQ BP Romanian DL Shoulder press Bent-over row Upright row	Volume load RPE Blood lactate Session duration Creatine kinase Training efficiency
Bentes et al. [52]	13 (M) 20 ± 1.3 years	RT: At least 5 years	AAS: 3 sets × failure Intraset rest: Succession Interset rest: 2 min TS: 3 sets × failure Interset rest: 2 min	10 RM	Chest press Low row Leg extension Leg curl Pull down Shoulder press	Volume load Blood pressure
Kelleher et al. [53]	10 (M) 21.7 ± 2.1 years	RT: At least 0.5 years	AAS: 4 sets × failure Intraset rest: Succession Interset rest: 1 min TS: 4 sets × failure Interset rest: 1 min	70% 1 RM	BP Bent over row Biceps curls Lying triceps extension Leg extension Leg curl	Energy cost rate Blood lactate
Gahreman et al. [48]	15 (unclear) 15.8 ± 1.0 years	RT: At least 3 years DL: 1.5 × bodyweight (1 RM) SQ: 1.9 × bodyweight (1 RM)	SS: 2 sets × 3 Intraset rest: Succession Interset rest: 2.5 min TS: 4 sets × 3 Interset rest: 2.5 min	90% 1 RM	SQ DL	
Paz et al. [54]	13 (M) 26.2 ± 3.9 years	RT: At least 5 years	AAS: 3 sets × failure Intraset rest: Succession Interset rest: 3 min TS: 3 sets × failure Interset rest: 1.5 min	10 RM	BP Lat pulldown 45° BP Seated close-grip row Triceps extension Biceps curl	Total number of repetitions Volume load Session duration Blood pressure
Wallace et al. [33]	11 (M) 24 ± 4 years	RT: 6 ± 5 years BP: 106 ± 42 kg (10 RM)	SS: 5 sets × ≤ 10 Intraset rest: Succession Interset rest: 2 min TS: 5 sets × ≤ 10 Interset rest: 2 min	10 RM	BP Incline BP	Volume load RPE Perceived recovery Acute muscle swelling Blood lactate Surface Electromyography
Paz et al. [55]	15 (M) 22.4 ± 1.1 years	RT: 3.5 ± 1.2 years	AAS: 3 sets × failure Intraset rest: 10 s Interset rest: 2 min TS: 3 sets × failure Interset rest: 2 min	10 RM	BP Wide-grip seated row	Volume load Session duration
Realzola et al. [56]	18 (9 F + 9 M) M: 24.1 ± 3.7 years F: 22.8 ± 3.9 years	RT: At least 1 year	AAS: 4 sets × failure Intraset rest: Succession Interset rest: 1 min TS: 4 sets × failure Interset rest: 1.5 min	75% 10 RM	Hexagonal bar DL Leg press Chest press Seated row Overhead dumbbell press Lat dorsi pull downs	RPE Session duration Energy cost rate Blood lactate
Garcia-Orea et al. [32]	17 (M) 23.9 ± 5.3 years	RT: Moderate strength-trained SQ: 93.6 ± 19.1 kg (1 RM) BP: 71.9 ± 12.4 kg (1 RM)	AS: 3 sets × VL 15% and 20% Intraset rest: 45 s Interset rest: 2 min TS: 3 sets × VL 15% and 20% Interset rest: 3 min	55–70% 1 RM	SQ BP	1 RM Session duration Total number of repetitions
Fink et al. [46]	23 (13 M + 1 0F) 18–23 years old	Athletes from a university gymnastics club	AAS: 3 sets × failure Intraset rest: Succession Interset rest: 1 min TS: 3 sets × failure Interset rest: 1 min	50–60% 1 RM	Biceps curls Overhead triceps extensions	Muscle CSA 1 RM MNR
Paz et al. [57]	22 (M) 25.2 ± 4.1 years	RT: 6.2 ± 5.2 years	AAS: 3 sets × failure Intraset rest: Succession Interset rest: 3 min TS: 3 sets × failure Interset rest: 1.5 min	10 RM	BP Lat pulldown 45° incline BP Seated close-grip row Triceps extension Biceps curl	Total number of repetitions Volume load Blood lactate Creatine kinase Surface Electromyography
Carregaro et al. [50]	14 (M) 29.4 + 6.1 years	Regular participation in aerobic exercise	AAS: 3 sets × 10 Intraset rest: Succession Interset rest: 1 min TS: 3 sets × 10 Interset rest: 1 min Unbalance training volume	60 and 180° s^−1^	Knee flexion Knee extension	
Merrigan et al. [47]	32 (F) 21 ± 2 years	Participation in a variety of recreational physical activities	SS: 3–4 sets × 8–12 Intraset rest: Succession Interset rest: 2.3–2.5 min TS: 3–4 sets × 8–12 Interset rest: 1 min	70–80% 1 RM	SQ Leg press	1 RM MNR Muscle CSA
Paz et al. [58]	14 (M) 22.2 ± 2.3 years	RT: 4.5 ± 1.2 years	AAS: 3 sets × failure Intraset rest: Succession Interset rest: 2 min TS: 3 sets × failure Interset rest: 2 min	8 RM	BP Seated row	Volume load Blood pressure

*AAS* agonist–antagonist superset, *AS* alternate peripheral superset, *BP* bench press, *CSA* cross-sectional area, *DL* deadlift, *F* female, *M* male, *MNR* maximal number of repetitions, *RPE* rating of perceived exertion, *RT* resistance training, *S* superset, *SQ* squat, *SS* similar biomechanical superset, *TS* traditional set, *VL* velocity loss, *1 RM* one repetition maximum

Moreover, due to their unbalanced training design, three studies were excluded from the meta-analysis [31, 50, 51]. For example, in the study by Antunes et al. [51], the superset group performed three sets of knee flexions and knee extensions to failure (totaling six sets), while the traditional set group only performed three sets of knee flexions to failure. This unbalanced training design may lead to difficulty in distinguishing whether the observed differences in acute and chronic effects arise from training volume or set structure.

### Risk of Bias Assessment

**Fig. 2:**
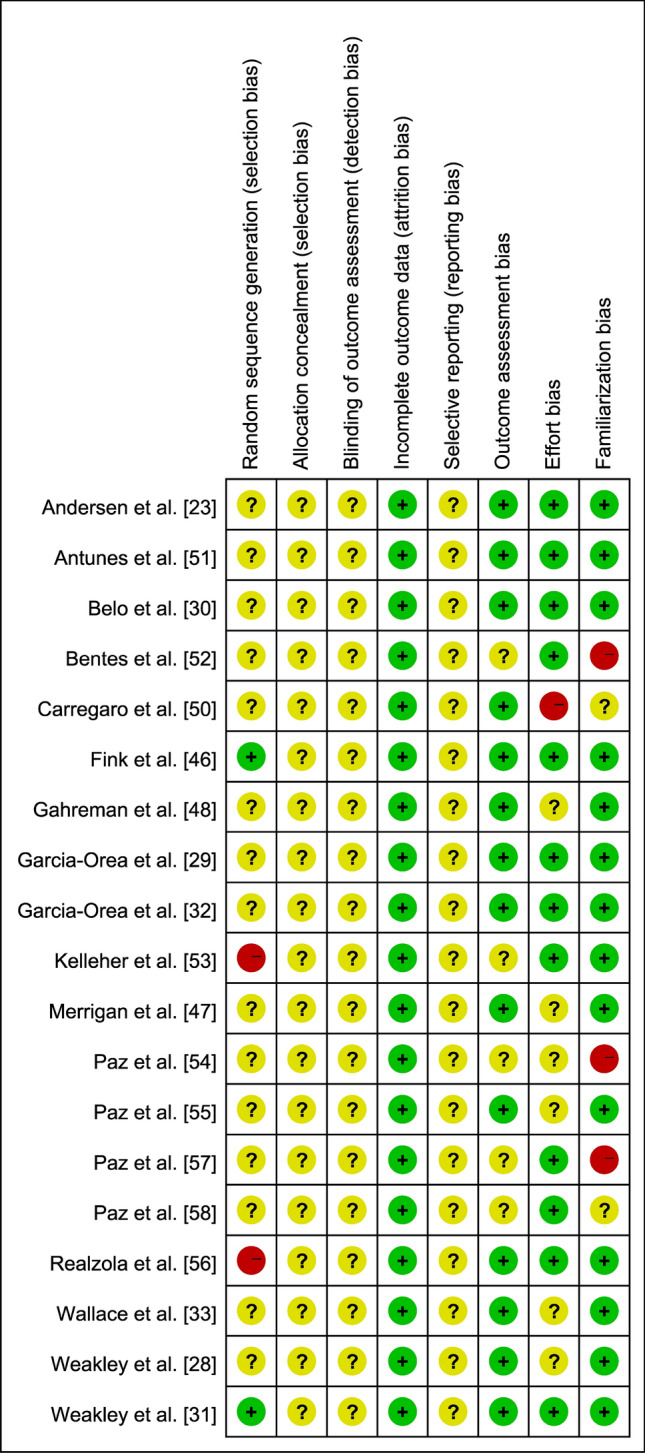
Risk of bias assessment (green circles represent low risk, yellow circles represent unclear risk, and red circles represent high risk)

On the basis of the assessment of the risk of selection bias, two studies were categorized as high risk of an order effect due to having fixed starting conditions [53, 56] (Fig. 2). Two studies were categorized as at low risk of an order effect [31, 46], while the remaining studies were classified as unclear risk. Moreover, no studies provided information on allocation concealment. In terms of detection bias, no studies reported information on outcome blinding procedures. Regarding attrition bias, no studies reported any missing outcomes. In terms of reporting bias, no studies preregistered their protocols, thus it remains unclear whether there is a risk of selective reporting. In the aspect of outcome assessment bias, five studies were categorized as unclear risk due to at least one device for which the validity could not be determined [52–54, 57, 58]. The rest were categorized as low risk. Regarding effort bias, one study was categorized as high risk because it did not involve participants performing exercises with a consistent effort level [50], six studies were categorized as unclear risk [28, 33, 47, 48, 54, 55], while the remaining studies were categorized as at low risk of effort bias. In the aspect of familiarization bias, three studies were categorized as high risk because they did not include any familiarization session or state that participants were familiar with all exercises used in the intervention [52, 54, 57]. Two studies were categorized as unclear risk [50, 58], while the remaining studies were categorized as at low risk of familiarization bias.

### Meta-analysis

#### Acute Effects

##### Mechanical Variables

*Total Number of Repetitions* There was no significant difference between superset and traditional set structures in total number of repetitions [SMD =  − 0.03 (95% CI  − 0.56 to 0.51); *p* = 0.92] (Table 2). However, subgroup analyses demonstrated that agonist–antagonist supersets resulted in a significantly greater total number of repetitions than traditional sets [SMD = 0.68 (95% CI 0.20 to 1.17); *p* = 0.01], but no significant difference was detected between alternate peripheral superset and traditional set prescriptions [SMD =  − 0.46 (95% CI  − 1.08 to 0.15); *p* = 0.14].

**Table 2:** Summary of the meta-analysis and quality of evidence synthesis of mechanical variables

Outcome	Meta-analysis							Grade				
	*k*	SMD		95% CI	95% PI	*p*-Value	*I*^2^	1	2	3	4	Quality
Total number of repetitions												
Supersets	6	− 0.03		− 0.56 to 0.51	− 1.69 to 1.64	0.92	66.9%	None	None	None	None	High
Agonist–antagonist supersets	2	0.68		0.20 to 1.17	k < 3	0.01	0%	− 1	− 1	None	None	Low
Alternate peripheral supersets	3	− 0.46		− 1.08 to 0.15	− 5.88 to 4.95	0.14	27.5%	None	− 1	None	None	Moderate
Volume load												
Supersets	8	0.05		− 0.48 to 0.57	− 1.71 to 1.81	0.86	78.4%	None	None	− 1	None	Moderate
Agonist–antagonist supersets	5	0.53		− 0.08 to 1.13	− 1.56 to 2.61	0.09	69.8%	− 1	None	None	None	Moderate
Similar biomechanical supersets	2	− 1.08		− 1.72 to − 0.44	*k* < 3	< 0.01	0%	None	− 1	None	None	Moderate
Alternate peripheral supersets	1	–		–	–	–	–	–	–	–	–	–
Training efficiency												
Supersets	2	1.74		0.46 to 3.01	*k* < 3	0.01	73.0%	None	− 1	None	None	Moderate
Alternate peripheral supersets	1	–		–	–	–	–	–	–	–	–	–

*CI* confidence interval, *k* number of trials, *PI* prediction intervals, *SMD* standardized mean differences (a positive SMD indicates higher values for supersets while a negative SMD indicates higher values for traditional sets), *1* risk of bias, *2* imprecision, *3* inconsistency, *4* indirectness

*Volume Load* The pooled analysis demonstrated no significant difference between superset and traditional set settings in volume load [SMD = 0.05 (95% CI  − 0.48 to 0.57); *p* = 0.86] (Table 2). Moreover, subgroup analyses showed that similar biomechanical supersets led to significantly less volume load than traditional sets [SMD =  − 1.08 (95% CI  − 1.72 to − 0.44); *p* < 0.01], but no significant difference was observed between agonist–antagonist superset and traditional set structures [SMD = 0.53 (95% CI  − 0.08 to 1.13); *p* = 0.09].

*Training Efficiency* The synthesized analysis revealed that supersets exhibited significantly higher training efficiency compared with traditional sets [SMD = 1.74 (95% CI 0.46 to 3.01); *p* = 0.01] (Table 2).

##### Metabolic Variables

*Blood Lactate Concentration during RT* The pooled analysis revealed that supersets led to significantly higher blood lactate concentration during RT compared with traditional sets [SMD = 0.94 (95% CI 0.08 to 1.81); *p* = 0.03] (Table 3). Furthermore, no significant differences were detected between the similar biomechanical superset and traditional set prescriptions [SMD = 0.34 (95% CI  − 0.42 to 1.10); *p* = 0.38].

**Table 3:** Summary of the meta-analysis and quality of evidence synthesis of metabolic variables

Outcome	Meta-analysis							Grade				
	*k*	SMD		95% CI	95% PI	*p*-value	*I*^2^	1	2	3	4	Quality
Blood lactate concentration during RT												
Supersets	4	0.94		0.08 to 1.81	− 2.78 to 4.66	0.03	72.3%	None	− 1	None	None	Moderate
Agonist–antagonist supersets	1	–		–	–	–	–	–	–	–	–	–
Similar biomechanical supersets	2	0.34		− 0.42 to 1.10	*k* < 3	0.38	37.0%	None	− 1	None	None	Moderate
Blood lactate concentration after RT												
Supersets	7	1.13		0.42 to 1.84	− 1.22 to 3.49	< 0.01	79.6%	None	None	− 1	None	Moderate
Agonist–antagonist supersets	3	1.52		0.04 to 3.00	− 16.70 to 19.73	0.04	89.1%	− 1	None	− 1	None	Low
Similar biomechanical supersets	2	0.30		− 0.30 to 0.90	*k* < 3	0.32	0%	None	− 1	None	None	Moderate
Alternate peripheral supersets	1	–		–	–	–	–	–	–	–	–	–
Creatine kinase concentration after RT												
Supersets	2	0.22		− 0.25 to 0.68	*k* < 3	0.36	0%	None	− 1	None	None	Moderate
Agonist–antagonist supersets	1	–		–	–	–	–	–	–	–	–	–
Energy cost during RT												
Supersets	2	1.93		0.08 to 3.78	*k* < 3	0.04	83.3%	− 1	− 1	− 1	None	Very low
Agonist–antagonist supersets	2	1.93		0.08 to 3.78	*k* < 3	0.04	83.3%	− 1	− 1	− 1	None	Very low
Surface electromyography												
Supersets	3	0.01		− 0.41 to 0.42	− 2.70 to 2.72	0.98	0%	None	− 1	None	None	Moderate
Agonist–antagonist supersets	1	–		–	–	–	–	–	–	–	–	–
Similar biomechanical supersets	2	0.01		− 0.58 to 0.60	*k* < 3	0.97	0%	None	− 1	None	None	Moderate
Acute muscle swelling												
Supersets	2	− 0.28		− 0.87 to 0.32	*k* < 3	0.36	0%	None	− 1	None	None	Moderate
Similar biomechanical supersets	2	− 0.28		− 0.87 to 0.32	*k* < 3	0.36	0%	None	− 1	None	None	Moderate
Systolic blood pressure												
Supersets	3	0.08		− 0.36 to 0.53	− 2.78 to 2.95	0.71	0%	− 1	− 1	None	None	Low
Agonist–antagonist supersets	3	0.08		− 0.36 to 0.53	− 2.78 to 2.95	0.71	0%	− 1	− 1	None	None	Low
Diastolic blood pressure												
Supersets	3	− 0.05		− 0.52 to 0.42	− 3.57 to 3.47	0.85	11.3%	− 1	− 1	None	None	Low
Agonist–antagonist supersets	3	− 0.05		− 0.52 to 0.42	− 3.57 to 3.47	0.85	11.3%	− 1	− 1	None	None	Low
Mean arterial pressure												
Supersets	3	− 0.03		− 0.48 to 0.41	− 2.89 to 2.82	0.88	0%	− 1	− 1	None	None	Low
Agonist–antagonist supersets	3	− 0.03		− 0.48 to 0.41	− 2.89 to 2.82	0.88	0%	− 1	− 1	None	None	Low

*CI* confidence interval, *k* number of trials, *PI* prediction intervals, *SMD* standardized mean differences (a positive SMD indicates higher values for supersets, while a negative SMD indicates higher values for traditional sets), *1* risk of bias, *2* imprecision, *3* inconsistency, *4* indirectness

*Blood Lactate Concentration after RT* The synthesized analysis demonstrated that supersets resulted in significantly higher blood lactate concentration after RT compared with traditional sets [SMD = 1.13 (95% CI 0.42 to 1.84); *p* < 0.01] (Table 3). Regarding subgroup analyses, agonist–antagonist supersets resulted in significantly higher blood lactate concentration than traditional sets [SMD = 1.52 (95% CI 0.04 to 3.00); *p* = 0.04]. However, no significant difference was observed between similar biomechanical superset and traditional set structures [SMD = 0.30 (95% CI  − 0.30 to 0.90); *p* = 0.32].

*Creatine Kinase Concentration after RT* There was no significant difference between superset and traditional set prescriptions in creatine kinase concentration after RT [SMD = 0.22 (95% CI  − 0.25 to 0.68); *p* = 0.36] (Table 3).

*Energy Cost during RT* The pooled analysis revealed a significantly higher energy cost during RT in supersets compared with traditional sets [SMD = 1.93 (95% CI 0.08 to 3.78); *p* = 0.04] (Table 3). The same result was also observed in the agonist–antagonist superset subgroup [SMD = 1.93 (95% CI 0.08 to 3.78); *p* = 0.04].

*Surface Electromyography* There was no significant difference between superset and traditional set settings in surface electromyography [SMD = 0.01 (95% CI  − 0.41 to 0.42); *p* = 0.98] (Table 3). Similarly, no significant difference was observed in the similar biomechanical superset subgroup [SMD = 0.01 (95% CI  − 0.58 to 0.60); *p* = 0.97].

*Acute Muscle Swelling* The pooled analysis demonstrated no significant difference between superset and traditional set structures in acute muscle swelling [SMD =  − 0.28 (95% CI  − 0.87 to 0.32); *p* = 0.36] (Table 3). Subgroup analyses also indicated no significant differences between similar biomechanical superset and traditional set prescriptions [SMD =  − 0.28 (95% CI  − 0.87 to 0.32); *p* = 0.36].

*Blood Pressure* The synthesized analysis did not reveal significant differences in systolic blood pressure [SMD = 0.08 (95% CI  − 0.36 to 0.53); *p* = 0.71], diastolic blood pressure [SMD =  − 0.05 (95% CI  − 0.52 to 0.42); *p* = 0.85], and mean arterial pressure [SMD =  − 0.03 (95% CI  − 0.48 to 0.41); *p* = 0.88] between superset and traditional set structures (Table 3). Subgroup analysis also showed consistent results in the agonist–antagonist superset subgroup.

##### Perceptual Variables

*Rating of Perceived Exertion* The pooled analysis demonstrated that supersets led to a significantly higher rating of perceived exertion than traditional sets [SMD = 0.77 (95% CI 0.15 to 1.40); *p* = 0.02] (Table 4). However, no significant difference was detected between similar biomechanical supersets and traditional set structures [SMD =  − 0.10 (95% CI  − 0.75 to 0.55); *p* = 0.75].

**Table 4:** Summary of the meta-analysis and quality of evidence synthesis of perceptual variables

Outcome	Meta-analysis							Grade				
	*k*	SMD		95% CI	95% PI	*p*-Value	*I*^2^	1	2	3	4	Quality
Rating of perceived exertion												
Supersets	6	0.77		0.15 to 1.40	− 1.29 to 2.84	0.02	74.80%	None	None	None	None	High
Agonist–antagonist supersets	1	–		–	–	–	–	–	–	–	–	–
Similar biomechanical supersets	2	− 0.10		− 0.75 to 0.55	*k* < 3	0.75	15.90%	None	− 1	None	None	Moderate
Alternate peripheral supersets	1	–		–	–	–	–	–	–	–	–	–
Perceived recovery												
Supersets	2	0.32		− 0.32 to 0.95	*k* < 3	0.33	11.60%	None	− 1	None	None	Moderate
Similar biomechanical supersets	2	0.32		− 0.32 to 0.95	*k* < 3	0.33	11.60%	None	− 1	None	None	Moderate

*CI* confidence interval, *k* number of trials, *PI* prediction intervals, *SMD* standardized mean differences (a positive SMD indicates higher values for supersets, while a negative SMD indicates higher values for traditional sets), *1* risk of bias, *2* imprecision, *3* inconsistency, *4* indirectness

*Perceived Recovery *There was no significant difference in perceived recovery between superset and traditional set settings [SMD = 0.32 (95% CI  − 0.32 to 0.95); *p* = 0.33] (Table 4). The same result was also observed in the similar biomechanical superset subgroup [SMD = 0.32 (95% CI  − 0.32 to 0.95); *p* = 0.33].

#### Chronic Variables

The pooled analysis demonstrated that supersets induced a similar chronic adaptation in maximal strength [SMD = 0.10 (95% CI  − 0.40 to 0.60); *p* = 0.36], strength endurance [SMD = 0.07 (95% CI  − 0.51 to 0.68); *p* = 0.81], and muscular hypertrophy [SMD =  − 0.05 (95% CI  − 0.63 to 0.54); *p* = 0.87] compared with traditional sets (Table 5).

**Table 5:** Summary of the meta-analysis and quality of evidence synthesis of chronic variables

Outcome		Meta-analysis					Grade				
	*k*	SMD	95% CI	95% PI	*p*-Value	*I*^2^	1	2	3	4	Quality
1 RM											
Supersets	3	0.10	− 0.40 to 0.60	− 3.14 to 3.35	0.36	0%	None	− 1	None	None	Moderate
Agonist–antagonist supersets	1	–	–	–	–	–	–	–	–	–	–
Similar biomechanical supersets	1	–	–	–	–	–	–	–	–	–	–
Alternate peripheral supersets	1	–	–	–	–	–	–	–	–	–	–
Maximal number of repetitions											
Supersets	2	0.07	− 0.51 to 0.68	k < 3	0.81	0%	None	− 1	None	None	Moderate
Agonist–antagonist supersets	1	–	–	–	–	–	–	–	–	–	–
Similar biomechanical supersets	1	–	–	–	–	–	–	–	–	–	–
Muscle CSA											
Supersets	2	− 0.05	− 0.63 to 0.54	k < 3	0.87	0%	None	− 1	None	None	Moderate
Agonist–antagonist supersets	1	–	–	–	–	–	–	–	–	–	–
Similar biomechanical supersets	1	–	–	–	–	–	–	–	–	–	–

*CI* confidence interval, *CSA* cross-sectional area, *k* number of trials, *PI* prediction interval, *SMD* standardized mean differences (a positive SMD indicates higher values for supersets while a negative SMD indicates higher values for traditional sets), *1* risk of bias, *2* imprecision, *3* inconsistency, *4* indirectness, *1 RM* one repetition maximum

## Discussion

**Fig. 3:**
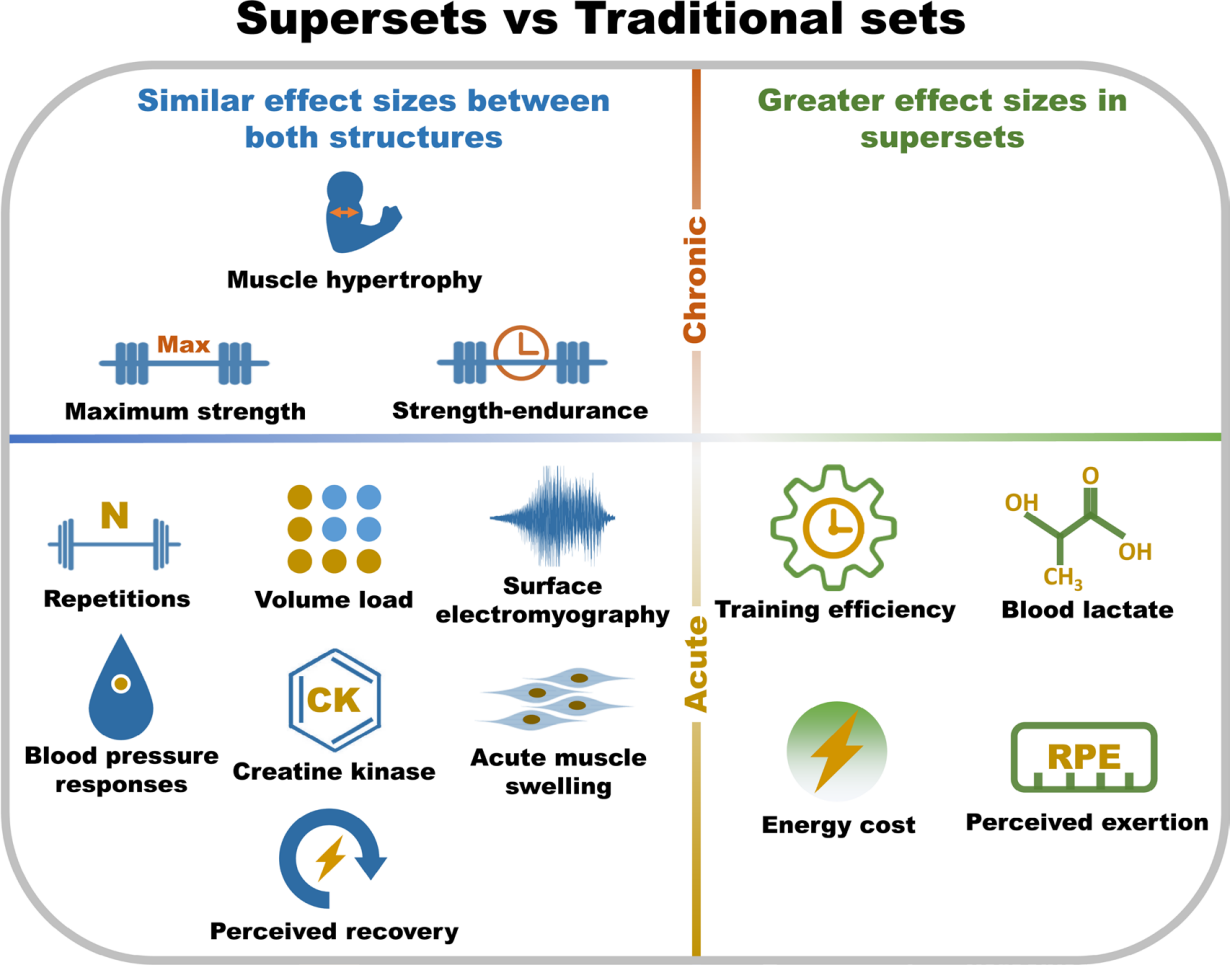
Comparison of acute responses and chronic adaptations between superset and traditional set prescriptions (*N*, total number of repetitions; CK, creatine kinase; RPE, rating of perceived exertion)

This systematic review and meta-analysis is the first to compare the acute and chronic effects of traditional and superset RT prescription on mechanical, metabolic, and perceptual variables. The findings from this review demonstrate that (1) superset prescription can maintain a similar total number of repetitions and volume load as traditional set prescription while enhancing training efficiency by reducing session duration; (2) superset prescription induces higher blood lactate concentrations and energy cost than traditional set prescription, but similar creatine kinase concentrations, surface electromyography, acute muscle swelling, and blood pressure responses; (3) supersets lead to higher perceived exertion than traditional set prescription, but similar perceived recovery; (4) superset prescription may induce similar long-term adaptations in maximal strength, strength endurance, and muscle hypertrophy as traditional set prescription (Fig. 3). Considering these findings, supersets should be recommended when individuals wish to increase training efficiency without impairing maximal strength, strength endurance, and muscle hypertrophy adaptations. However, it is important to note that supersets can increase the metabolic response and perception of effort during training. Consequently, when using supersets, greater recovery between sessions may be prudent and the placement of this form of training should be carefully considered within a periodized program.

### Acute Variables

#### Training Volume and Efficiency

Our findings showed that supersets reduced training time by approximately 37% when compared with traditional set structures. This is primarily due to superset prescription decreasing rest frequency or duration of rest periods. For example, in the study by Paz et al. [55], participants performed three sets of bench press and seated row. In the traditional set condition, participants rested five times (e.g., 2 min each time; 10 min in total) between sets and exercises, while the superset condition only involved two rest intervals (e.g., 2 min each; 4 min in total) because the two exercises of the superset were performed without rest. Additionally, although superset structures sacrifice some rest time, this does not necessarily result in a decreased training volume. Our point estimates revealed negligible differences between superset and traditional set prescriptions in the total number of repetitions (SMD =  − 0.03; *p* = 0.92) and volume load (SMD = 0.05; *p* = 0.86). However, there is some uncertainty due to the wide prediction intervals [total number of repetitions (95% PI: − 1.69 to 1.64) and volume load (95% PI: − 1.71 to 1.81)]. On the other hand, superset prescription has higher training efficiency than traditional set structures (SMD = 1.74; *p* = 0.01) because the same training volume was completed in less training time. Considering this change in training efficiency, it could easily be hypothesized that this decrease in interset rest would lead to greater fatigue accumulation, which would acutely impair strength and thereby reduce training volume [59]. However, supersets can mitigate localized muscle fatigue responses through the strategic selection of different exercises [31]. In this review, most studies (*n* = 16) that implemented supersets used two RT exercises targeting different muscle groups, which helped avoid continuous work on the same muscle group and allowed for the maintenance of training volume. Consequently, practitioners should use supersets during time-constrained periods (e.g., when athletes are balancing intense training and competition schedules or office workers with limited time for exercise) and can be confident that similar training volumes can be completed within a shorter timeframe.

Our subgroup analysis revealed that agonist–antagonist supersets enabled individuals to complete more repetitions compared with traditional sets (SMD = 0.68; *p* = 0.01). This may be because antagonist preloading potentially facilitates increased neural activation, which acutely enhances strength performance and thereby allows for a higher training volume [55, 60]. Thus, this type of superset is most suitable for athletes who wish to complete a large training volume in a short amount of time. In contrast, similar biomechanical supersets resulted in less training volume than traditional sets (SMD =  − 1.08; *p* < 0.01). Therefore, similar biomechanical supersets should be avoided unless individuals are required to train in close proximity to failure, which may be useful for the development of muscle hypertrophy.

#### Internal Load

Although there was considerable variance in blood lactate responses [blood lactate concentration during RT (95% PI: − 2.78 to 4.66) and blood lactate concentration after RT (95%PI: − 1.22 to 3.49)], the point estimates demonstrated that supersets led to higher blood lactate concentration during (SMD = 0.94; *p* = 0.03) and after RT (SMD = 1.13; *p* < 0.01) compared with traditional sets, along with a higher energy cost (SMD = 1.93; *p* = 0.04). In addition, studies by Realzola et al. [56] and Weakley et al. [28] showed that supersets also induced higher heart rate, oxygen consumption, and endogenous testosterone responses when compared with traditional set prescription. This is primarily because supersets often require individuals to perform a similar training volume as traditional sets but in a shorter training time. Consequently, coaches must be aware that supersets can impose higher internal loads and this may lead to more severe post-exercise fatigue and decreased athletic performance [61, 62]. Therefore, a longer recovery period could be needed following a session that implements supersets.

Our findings show that similar biomechanical supersets did not result in significantly higher blood lactate concentration during (SMD = 0.34; *p* = 0.38) and after RT (SMD = 0.30; *p* = 0.32) compared with traditional sets. Theoretically, similar biomechanical supersets, which impose repeated contractions of the same muscle group, would lead to higher blood lactate concentration [63]. However, this expected outcome was not observed in our subgroup analysis. In practice, the impact of RT on blood lactate concentration is influenced by various training settings such as exercise selection, loading intensity, and volume [64–66]. In this review, the study that investigated the impact of similar biomechanical supersets on blood lactate had a substantially lower training volume (e.g., 2 exercises × 5 sets) [33] compared with other forms of supersets (e.g., 4–6 exercises × 3–4 sets) [28, 30, 53, 56, 57]. This low training volume may have resulted in only minor changes in blood lactate concentration [67, 68]. Consequently, while further volume- and time-equated research that investigates the internal response to similar biomechanical supersets is warranted, it is likely that utilizing low training volumes could be an effective strategy for mitigating the high lactate concentrations often induced through supersets.

#### Muscle Damage

The pooled analyses demonstrated that supersets did not result in significantly higher muscle damage when compared with traditional sets [creatine kinase concentration (SMD = 0.22; *p* = 0.36) and acute muscle swelling (SMD =  − 0.28; *p* = 0.36)]. In this review, three studies investigated the impact of supersets on proxies of muscle damage, with two studies reporting similar muscle damage between superset and traditional set prescriptions [33, 57]. Of note, Weakley et al. [28] indicated that supersets resulted in more severe muscle damage compared to traditional sets, as evidenced by higher creatine kinase concentration. This discrepancy in results is interesting as supersets lead to higher internal loads, and it is plausible that the greater internal loads experienced by individuals who implement supersets would lead to more severe muscle damage [69, 70]. One reason for these contrasting findings could be due to the training interventions employed. For example, one of the studies that reported similar muscle damage used a longer rest time within the superset condition (e.g., 3 min rest between supersets versus 1.5 min rest between traditional sets) [57]. Such extended recovery times likely mitigate muscle damage induced by RT [71]. Additionally, the other study that found similar muscle damage outcomes employed relatively low training volumes (e.g., 2 exercises × 5 sets) [33], while Weakley et al. [28] found the opposite result when applying a higher training volume (e.g., 6 exercises × 3 sets) within a reduced rest time. This difference in total training volume and efficiency may have contributed to the variation in observed muscle damage. Thus, when using supersets, implementing extended recovery times and low training volumes may serve as effective strategies to alleviate muscle damage.

#### Muscle Activation

Our point estimate suggested no significant difference in surface electromyography between superset and traditional set structures (SMD = 0.01; *p* = 0.98), with notable variability observed in this finding (95% PI: − 2.70 to 2.72). Thus, RT using superset prescription may result in similar muscle activation as traditional set prescription, but this result comes with a degree of uncertainty. Furthermore, previous studies have suggested that antagonist preloading contributes to inducing higher muscle activation [55, 60]. However, Paz et al. [57] examined the impact of agonist–antagonist supersets on muscle activation and found that agonist–antagonist supersets induced similar or even lower muscle activation in local muscles compared with traditional sets. The authors claimed that fatigue might be the reason for this phenomenon, as they applied a much higher training volume (e.g., 6 exercises × 3 sets) than typical investigations of antagonistic activation (e.g., 1–2 exercises × 2–4 sets) [72, 73]. Therefore, coaches need to be aware that the proposed advantages of agonist–antagonist supersets in inducing high muscle activation may be attenuated by fatigue.

#### Blood Pressure

Future effect sizes in blood pressure remain uncertain, as evidenced by the wide prediction intervals [systolic blood pressure (95% PI: − 2.78 to 2.95), diastolic blood pressure (95% PI: − 3.57 to 3.47), and mean arterial pressure (95% PI: − 2.89 to 2.82)]. However, our point estimates suggested that superset prescription resulted in similar blood pressure responses to traditional set prescription [systolic blood pressure (SMD = 0.08; *p* = 0.71), diastolic blood pressure (SMD =  − 0.05; *p* = 0.85), and mean arterial pressure (SMD =  − 0.03; *p* = 0.88)]. Traditionally, RT has been recognized as an effective nonpharmacological approach for enhancing cardiovascular health [74, 75]. One reason for this is that RT can induce post-exercise hypotension (i.e., an acute reduction in blood pressure during the postexercise period to levels below the baseline values pre-workout) [76–78]. Two studies included in this review observed significant reductions in both systolic and diastolic blood pressure after superset RT, and these reductions persisted for over 50 min [54, 58]. Consequently, superset prescription may be comparable in eliciting post-exercise hypotension to traditional set prescriptions.

#### Perceived Exertion and Recovery

Our findings indicated that supersets resulted in greater perceived exertion compared with traditional set prescriptions (SMD = 0.77; *p* = 0.02), with a wide range of variability observed in this finding (95% PI: − 1.29 to 2.84). The elevated perceived exertion associated with supersets is not unexpected, given their shorter recovery time and enhanced training efficiency. However, although supersets result in greater exertion during RT, this may not always be captured in commonly used load monitoring tools [79]. Weakley et al. [28] investigated the effect of superset structures on session perceived load, which is calculated as the product of session duration and session rating of perceived exertion. They found that supersets had a lower session perceived load compared with traditional sets due to the shorter session duration. This indicates that although implementing superset RT may be perceived as harder for athletes, it is brief, and this may be a confounding factor in load monitoring systems that utilize both perceptual (e.g., rating of perceived exertion) and duration outcomes.

Superset prescription has a similar perceived recovery to traditional set prescription (SMD = 0.32; *p* = 0.33). In this review, two studies by Wallace et al. [33] examined the impact of superset RT on perceived recovery. They found that individuals completing five sets of bench press and incline bench press at 10 RM reported comparable perceived recovery between superset and traditional set prescriptions, despite the superset group having less rest time (i.e., a total of 8 min in the superset group versus a total of 19 min in the traditional set group). Therefore, under similar conditions, reduced rest time in supersets does not compromise athletes’ perceived recovery status.

Regarding the perceptual responses during different types of supersets, Weakley et al. [31] showed that similar biomechanical supersets induced the highest perceived exertion compared with agonist–antagonist and alternate peripheral supersets. This difference in perceptions of exertion could be attributed to the muscle groups used within each superset. Specifically, similar biomechanical supersets impose repeated contraction of the same muscle groups, leading to higher levels of fatigue and increased perceived exertion among individuals [80–82]. On the other hand, agonist–antagonist and alternate peripheral supersets involve consecutive RT exercises targeting different muscle groups, allowing better recovery of muscle groups and resulting in lower perceived exertion [31, 82]. Consequently, when implementing supersets, agonist–antagonist and alternate peripheral superset prescriptions may help individuals save time while avoiding a substantial increase in perceived exertion.

### Chronic Variables

There was considerable variance in estimates of future maximal strength adaptations (95% PI: − 3.14 to 3.35), but the point estimate suggested similarity between superset and traditional set configurations (SMD = 0.10; *p* = 0.36). Fink et al. [46] reported that superset prescription did not lead to significant maximal strength improvements after an 8-week intervention. This could be attributed to the focus of the study on low-intensity supersets using an intensity of approximately 55% 1 RM. Such an intervention may not provide the specific stimulus required to induce maximal strength improvement [83, 84]. According to the principle of specificity, the closer a training program aligns with the demands of a specific outcome, the more effectively it transfers to that outcome [85]. Therefore, it is logical that training closer to an individual’s 1 RM would yield greater transfer to maximal strength outcomes [2]. Thus, when implementing superset RT to enhance maximal strength, despite the increased training efficiency, there is still a need to carefully consider exercise specificity, particularly exercise load.

Two studies investigated the effects of superset prescription on muscle endurance and hypertrophy, with both their findings supporting the notion that supersets can induce similar chronic adaptations in strength endurance (SMD = 0.07; *p* = 0.81) and muscle hypertrophy (SMD =  − 0.05; *p* = 0.87) as traditional set prescription. Thus, supersets can be used in athletes’ periodized training programs when the purpose is to develop muscle endurance and/or hypertrophy while having limited training time. In addition, it is worth noting that a study by Fink et al. [46] reported that an 8-week superset RT intervention with low-intensity (50–60% 1 RM) is sufficient to induce strength endurance and hypertrophy adaptations. Therefore, if the training goal is solely to improve muscle endurance and/or hypertrophy, using low-intensity superset RT may improve efficiency and reduce the need for heavy loads.

### Limitations

Several limitations should be acknowledged when interpreting the findings of this review. First, the majority of individuals included in this systematic review and meta-analysis are young and trained males, which may potentially limit the generalizability of our findings to other populations, such as females, older individuals, and those who are untrained. Second, there may be confounding factors influencing our findings. For example, total training volume potentially influences the impact of supersets on internal loads, rest time potentially alters the influence of supersets on muscle damage, and RT intensity (as a percentage of the one-repetition maximum) potentially moderates the impact of supersets on strength adaptations. However, due to limited sample sizes for most of the outcomes, further subgroup analyses and meta-regression could not be conducted to identify potential moderators. Third, we conducted subgroup analyses to explore differences between different superset configurations (e.g., similar biomechanical versus alternate peripheral). However, the limited number of included studies only allowed subgroup analysis to be performed for certain outcomes. As a result, we are unable to provide a comprehensive understanding of how different types of supersets influence mechanical, metabolic, and perceptual responses. Finally, some outcomes were affected by high levels of heterogeneity. Although we conducted subgroup analyses, the source of heterogeneity cannot be fully determined. We speculate that the small number of included studies may be one of the main reasons contributing to the high levels of heterogeneity.

## Conclusions

The current systematic review and meta-analysis aimed to compare the acute and chronic effects of superset and traditional set prescriptions on mechanical, metabolic, and perceptual responses. Our findings suggest that supersets offer a time-efficient alternative to traditional RT, enabling individuals to reduce training time without compromising training volume, muscle activation, or perceived recovery. Moreover, individuals using supersets can achieve comparable chronic adaptations to traditional set prescription in maximal strength, strength endurance, and muscle hypertrophy. However, researchers, sports professionals, and athletes should note that individuals implementing superset RT will experience greater internal loads along with higher levels of muscle damage and perceived exertion. Thus, considering longer recovery time following superset RT sessions could be beneficial. Additionally, superset RT may have a similar potential to traditional RT in eliciting post-exercise hypotension. On the other hand, agonist–antagonist supersets are advantageous for maintaining training volume and may be more suitable for individuals who are required to perform more training volume in time constrained periods. Similar biomechanical supersets, while shortening training time, concentrate stimulation on the same muscle group, causing individuals to train in closer proximity to failure and potentially making them more suitable for individuals who want to increase muscle hypertrophy.

## Declarations

### Availability of Data and Material

The datasets generated and/or analyzed during the current review are available from the corresponding author on reasonable request.
